# Pressure measurement devices: from technical assessment to clinical performance

**DOI:** 10.1186/1757-1146-5-S1-P8

**Published:** 2012-04-10

**Authors:** Claudia Giacomozzi, Moreno D’Amico, Piero Roncoletta

**Affiliations:** 1 Dept. Of Technology and Health, Italian National Institute of Health (ISS), Rome, Italy; 2 Bioengineering and Biomedicine Company srl, Pescara, Italy

## Background

Technical assessment of pressure measurement devices (PMDs) should guarantee for their appropriate use in the clinics. The study aims at proving the validity of the assessment methodology ISS proposed [1], and at quantifying the impact of PMD performance on clinical assessment.

## Materials and methods

Three commercial PMDs were first assessed and then compared during barefoot walking: PMDa and PMDb - resistive technology, 1sens/cm^2^ – were assessed on-site, while PMDc – capacitive technology, 4sens/cm^2^ - was tested on-the-bench and on-site [1]. The PMDs were aligned on the floor to capture successive at-regimen steps of the left foot of one trained volunteer; 10 complete steps were acquired in both directions for each PMD; data were temporally normalised and averaged; main kinetic parameters were extracted.

## Results

**Figure 1:**
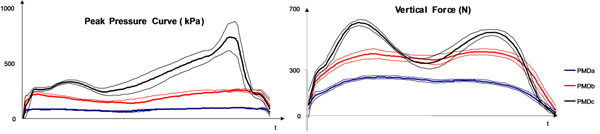
Peak Pressure and Vertical Force curves obtained by the three tested PMDs; mean curve ± sd curve averaged over 10 left steps.

Preliminary results (Table 1 and Figure 1): i) PMDc resulted accurate and was used as a reference; ii) PMDa was found inaccurate on-site and delivered unreliable gait data; iii) PMDb was found accurate on-site but performed significantly worse than PMDc during gait.

**Table 1:** Results from the on-the-bench and on-site assessment, and with respect to some clinically relevant parameters.

PMD under test	ISS Full technical assessment	ISS On-site partial assessment	“gait” assessment: Peak pressure (kPa)	“gait” assessment: Mean pressure (kPa))	“gait” assessment: Integral (kPa*s) [2]
a	not performed	error >10% at 250kPa	100 (4)**	80 (2)**	39 (2)**
b	not performed	error < 5% at 250kPa	266 (12)*	191 (8)*	85 (9)*
C	accuracy error < 5% up to 1200kPa	error < 5% at 250kPa	744 (137)	367 (17)	152 (23)

* statistically different from PMDc corresponding data (p<0.05, also verified with respect to the ± 5% maximum error); ** statistically different from PMDb and PMDc corresponding data (p<0.05, also verified with respect to the ± 5% maximum error)

## Conclusions

To conclude: i) on-site assessment up to 250kPa proved to be necessary but not sufficient to guarantee for a good PMD performance during gait; ii) a thorough on-the-bench assessment is effective and recommended; iii) use of PMDb data might be misleading in research and risky in the clinics. The study is going on with the comparison among other commercial PMDs and under a wide range of testing conditions.
